# A Special Material or a New State of Matter: A Review and Reconsideration of the Aerogel

**DOI:** 10.3390/ma6030941

**Published:** 2013-03-08

**Authors:** Ai Du, Bin Zhou, Zhihua Zhang, Jun Shen

**Affiliations:** 1 Shanghai Key Laboratory of Special Artificial Microstructure Materials and Technology, Tongji University, Shanghai 200092, China; E-Mails: zzhtj@tongji.edu.cn (Z.Z.); shenjun67@tongji.edu.cn (J.S.); 2 School of Physics Science and Engineering, Tongji University, Shanghai 200092, China

**Keywords:** aerogel, preparation, review, state of matter, nanoporous, sol-gel, history, tendency

## Abstract

The ultrahighly nanoporous aerogel is recognized as a state of matter rather than as a functional material, because of its qualitative differences in bulk properties, transitional density and enthalpy between liquid and gas, and diverse chemical compositions. In this review, the characteristics, classification, history and preparation of the aerogel were introduced. More attention was paid to the sol-gel method for preparing different kinds of aerogels, given its important role on bridging the synthetic parameters with the properties. At last, preparation of a novel single-component aerogel, design of a composite aerogel and industrial application of the aerogel were regarded as the research tendency of the aerogel state in the near future.

## 1. Introduction

**Figure 1:**
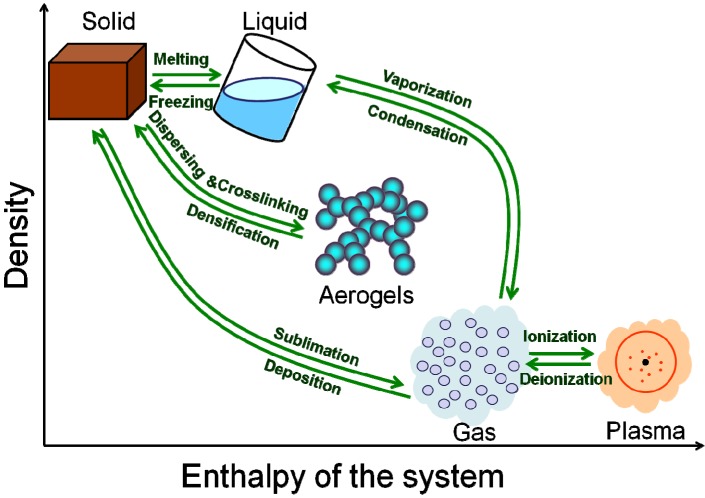
The distribution and transition of different states of matter in “density” *vs*. “enthalpy of the system” diagram.

As we know, there are three common states of matter: solid, liquid and gas. Even if adding a plasma state, you will find the density and enthalpy of the states are interrupted. As shown in Figure 1, the density of the solid and liquid (or gas and plasma) are much the same, but the density between liquid and gas differs by 3–4 orders of magnitude. In addition, the enthalpy of the system of liquid and gas states differs greatly. Why does the nature leave a huge gap between the liquid state and the gas state?

Unfortunately, the gap not only exists theoretically, but also affects human beings. For example, the acoustic impedance mismatch in the interface of piezoelectric transducer and gas results in energy loss of more than 90% [1]. The density difference between the solid and gas states of deuterium-tritium mixture may lead to the ignition failure in the inertial confinement fusion experiment [2,3]. In the laser–X-ray conversion experiment, the solid or liquid targets present extremely low efficiency (0.0001%~0.5%), while the X-ray wavelength conversed by the gas targets is limited by K-shell emission of only several kinds of inert gases [4,5,6,7]. Furthermore, in the Cherenkov detection experiment, a density-induced gap of refractive index makes high-momentum charged particles (>4 GeV/c) undetectable [8,9]. High-pressure fluid is supposed to solve these problems, but may cause much more technical difficulties.

As shown in Figure 1, the aerogel could, to a great extent, fill the gap between the liquid and gas state. Aerogel is a kind of material with three-dimensional open networks assembled by coherent NPs or polymer molecules [10,11,12,13]. Given the recent development, the aerogel could be recognized as not only a special functional material, but also a new state of matter [14]. On the one hand, the aerogel exhibits qualitative differences in bulk properties in comparison with other states of matter. Like the solid state, aerogel maintains a fixed volume and shape. However, the density of aerogel could range from 1000 kg/m^3^ above (solid density) to about 1 kg/m^3^ (lower than the density of the air), which induces dramatic changes in the properties. Due to not only high porosity like other foams, but also dual structural natures of microscopic (nanoscale skeleton) and macroscopic (condensed state matter) features, aerogel exhibits versatile unique properties such as ultralow thermal conductivity, ultralow modulus, ultralow sonic velocity, ultralow refractive index, ultralow dielectric constant, ultralow sound speed, high specific surface area and ultrawide adjustable ranges of the density and the refractive index [11,12,15,16].

On the other hand, “aerogel” state matter includes diverse composition as other states do. During about 70 years after the aerogel was invented (1931) [17], the aerogel research only focused on limited compositions such as silica, several kinds of non-silica oxides, resorcinol formaldehyde (RF) aerogel, carbonized-RF (CRF) aerogel and aerogel composites [12]. After entering the 21st century, aerogel category was booming. Lots of novel non-silica oxide aerogels [18,19,20,21,22,23,24,25,26,27,28], chalcogenide aerogels [29,30,31,32,33,34,35], gradient aerogels (from 90 s) and other aerogel composites sprang up one after another [36,37,38,39]. Recently, novel aerogels such as carbon nanotube (CNT) aerogel [40,41,42,43], graphene aerogel [44,45,46,47,48,49,50], silicon aerogel and carbide (or carbonitride) aerogel were added into the aerogel community continually [51,52,53,54,55]. It can be expected that, without exaggeration, hardly any substances could not be converted into the aerogel.

Thus, this paper will introduce the characteristics, classification, history, preparation and research tendency of the aerogel based on the point of treating the aerogel as a state of matter.

## 2. Characteristics, Classification and History

### 2.1. Basic Characteristics

There is no uniform definition of the term aerogel. In fact, this term is still developing. However, there is an important feature that almost all aerogels reported are derived from the wet gel via a sol-gel process. Therefore, the aerogel is unable to be defined without referring to the gel. The aerogel is defined by IUPAC (international union of pure and applied chemistry) as a “gel comprised of a microporous solid in which the dispersed phase is a gas” [56]. In the “Aerogel Handbook”, Pierre adopts the initial idea of Kistler to define it as the “gels in which the liquid has been replaced by air, with very moderate shrinkage of the solid network” [57]. This concept is simplified, appropriate and widely acceptable. A similar but longer definition in Hüsing’s review (also in Ullmann’s Encyclopedia of Industrial Chemistry) designates the aerogel as the “materials in which the typical structure of the pores and the network is largely maintained … while the pore liquid of a gel is replaced by air” [58].

However, the aerogel is increasingly recognized as a matter with special structure and characteristics, neglecting the preparation or drying method. Aerogel-related porous materials defined originally like xerogel and cryogel are gradually accepted as the aerogel. Even in some works, the aerogel is neither derived from the gel, nor underwent a sol-gel process. For example, the carbon nanotube aerogel were directly “drawn from straight sidewalls of multiwalled nanotube … forests that were synthesized by catalytic chemical vapor deposition” [43]. This indicates that the aerogel do not need to be a gel but a matter with gel-like structure. Thus, the concept of aerogel is suggested being regarded as a state of matter whose structure is similar to the solid networks of a gel with gas, or vacuum in-between the skeletons, considering the considerable progress. This definition, moreover, ensures the aerogel in a high vacuum environment could be still called “aerogel”.

An aerogel state matter should possess below two characteristics:

(1) Structure characteristic: gel-like structure, normally with nanoscale coherent skeletons and pores; hierarchical and fractal microstructure (primary structure coexists and is related with larger-scale structure); able to form macroscopic monolith; randomly crosslinking network, normally composed of non-crystalline matter.
(2) Property characteristic: unique bulk properties different from solid matter, gas matter or normal foam, such as ultralow thermal conductivity, ultralow modulus, ultralow refractive index, ultralow dielectric constant, ultralow sound speed, high specific surface area and ultrawide adjustable ranges of the density and the refractive index (especially for silica aerogel); ultralow relative density and ultrahigh porosity.

The structure of the aerogels could be characterized by using an electron microscope, pore size analyzer, small angle X-ray scattering and so on. The properties are normally measured by specific instruments. For example, the mechanical properties (stress-strain curve, strength, modulus and loss tangent) of the aerogels could be tested by accurate universal testing machine or dynamic thermomechanical analyzer in a compression or three-point bending mode.

According to this description, not all kinds of ultralight foams can be classified as the aerogel state. For example, the ultralight metallic microlattices do not belong to the aerogel state because they do not exhibit gel-like fractal microstructure [59].

### 2.2. Classification

There are several methods used to classify the aerogel. By considering its appearance, the aerogel could be divided into monolith, powder and film; and by considering the preparation method, aerogel could be made up of four types including aerogel (as defined by Pierre), xerogel, cryogel and other aerogel-related materials; while given the different microstructure, aerogel could be classified as microporous (<2 nm) aerogel, mesoporous (2~50 nm) aerogel and mixed-porous aerogel.

**Figure 2:**
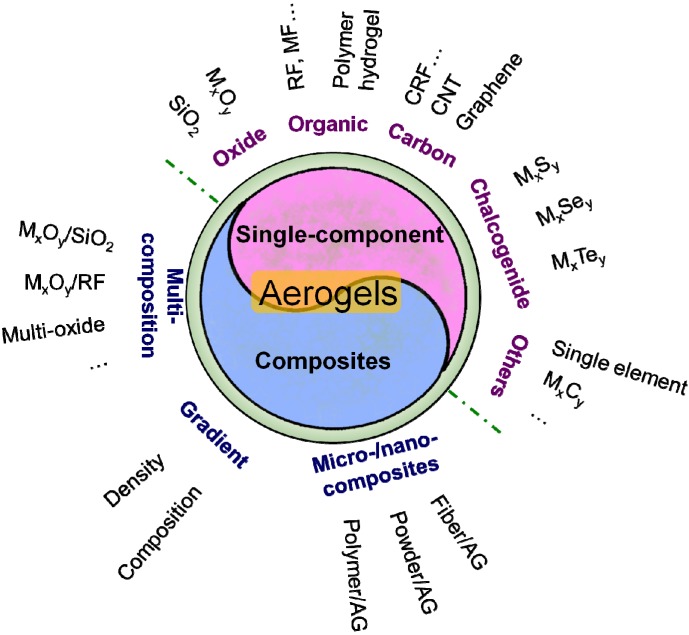
The classification of the aerogels.

However, the most acceptable viewpoint to classify the aerogel is to distinguish them by their composition. As shown in Figure 2, the aerogel could be divided into two categories: single-component aerogels and aerogel composites. Single-component aerogel consists of oxide aerogel (silica and non-silica), organic aerogel (resin-based and cellulose-based), carbon aerogel (carbonized plastic, CNT and graphene), chalcogenide aerogel and other kinds of aerogel (single element, carbide, *etc.*). Aerogel composite includes multi-composition aerogel, gradient aerogel and micro-/nano- aerogel composites.

### 2.3. Brief History

The aerogel was invented by Kistler in 1931. Its inventor named it as “aerogel” (air + gel) because it replaced the liquid component inside the wet gel with the air without damaging the solid microstructure [17]. Although this interesting material exhibited some fantastic properties, it seemed that the aerogel did not arouse broad interests before 1970. After that, the aerogel research became hotter and hotter. By the end of 2012, there are 3612 papers recorded in Science Citation Index (SCI), searching with the keyword “aerogel” as the topic. The average citations of papers published during 1931–2012 and 2008–2012 (recent five years) is 15.49 and 6.33, respectively. The Journal of Non-Crystalline Solids published more than 10% papers, while another five journals (all published more than 2% papers, including Nuclear Instruments Methods in Physics Research A, Journal of Sol-Gel Science and Technology, Journal of Low Temperature Physics, Physical Review Letter and Meteoritics Planetary Science) totally published about 15% papers. In the last five years, the Journal of Non-Crystalline Solids published ~6% papers, while another seven journals (>2% papers) published about 23% papers in total. It seems that more journals such as Journal of Materials Chemistry, Microporous and Mesoporous Materials and Journal of Physical Chemistry C are willing to receive the submissions about the aerogel. High average citations and wide distribution of frequent journals indicate that aerogel research has gotten sufficient and broad attention. Here the number of papers published every year, after searching in SCI, Engineering Index (EI) and Google Scholar, is used to study the development of the aerogel during 1994–2012, 1982–2012 and 1931–1985, respectively.

In the early stage, the aerogel (silica) was prepared via acid-catalyzed reaction with water glass, washing remove of chloride ion, solvent exchange from water to ethanol and supercritical fluid drying [60]. However, the time-consuming processes of washing and solvent exchange seem to be unacceptable for the other researchers. Before 1970, there are only one or two papers published occasionally in one year.

**Figure 3:**
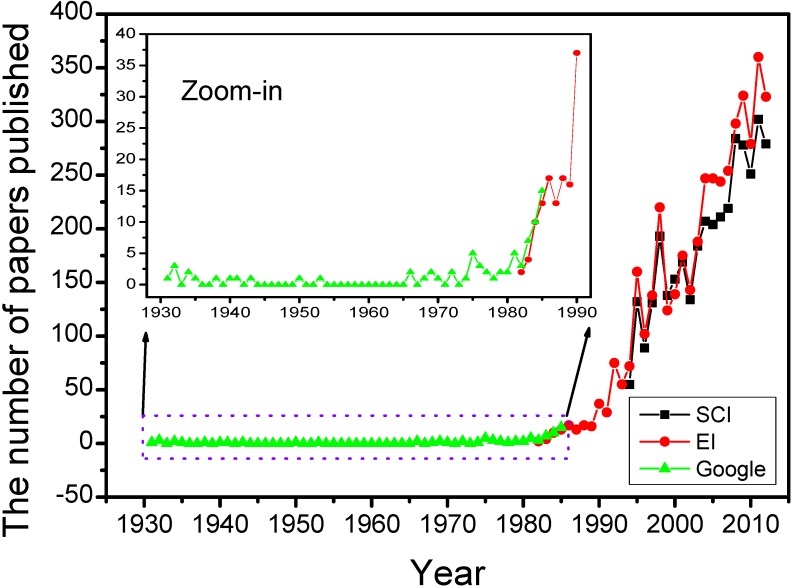
The number of papers published every year.

As shown in Figure 3, the aerogel research has undergone three upsurges after 1970. The first ones appeared in the period of the 1970s and 1980s. The significant innovation is the replacement of waterglass/water systems with organic precursor and corresponding organic solvent to prepare aerogels fast. The representative works are the usage of tetramethyorthosilicate (TMOS) by Teichner’s group in 1968, safer tetraethylorthosilicate (TEOS) by Russo *et al.* in 1986 [61,62], and the development of carbon dioxide supercritical fluid drying [63]. What is more important, the hydrolysis and condensation of the alkoxide are relatively simple and controllable, which means the formation mechanism was developed rapidly. The first and second international symposiums on aerogels (ISA) were held in 1985 and 1988, respectively. These were the earliest specialized conferences taking “aerogel” as the main topic, which greatly promoted the development of the aerogel.

The second upsurge occurred in 1990s. The significant affair was the birth of organic and carbon aerogel and the invention of surface-modified ambient drying [64,65,66,67]. Numerous intensive studies of applications and new attempts for industrialization were done, which made the aerogel become a competitive material, both in properties and the cost [57]. Furthermore, the third to sixth ISA continued to promote the aerogel research. Many potential applications discussed in these symposiums are even studied till now.

The third upsurge happened from the early 21st century to present, which was explosive and, to some extent, spontaneous. Many achievements were realized in this period. Gash developed a versatile method to prepare diverse oxide aerogels by using inorganic salt as precursor and epoxide as gelation accelerator in 2001 [68,69]. Brock invented the chalcogenide aerogel via reverse micelle synthesis, a sol-gel process and supercritical fluid drying in 2004 [35]. Gradient aerogels successfully captured ultrahigh-velocity particles from the comet and interstellar space, and returned to the earth in 2006 [38,39,70]. After that, a series of novel aerogels including CNT aerogel, graphene aerogel, carbide aerogel and single-element aerogel were created in succession [43,49,50,51,52,53]. In addition, the properties, applications and commercialization of the aerogel were widely developed in this period. More and more scientists, engineers, government officers and the public pay close attention to the aerogel field, which makes a bright future of the aerogel expectable.

## 3. Preparation

**Figure 4:**
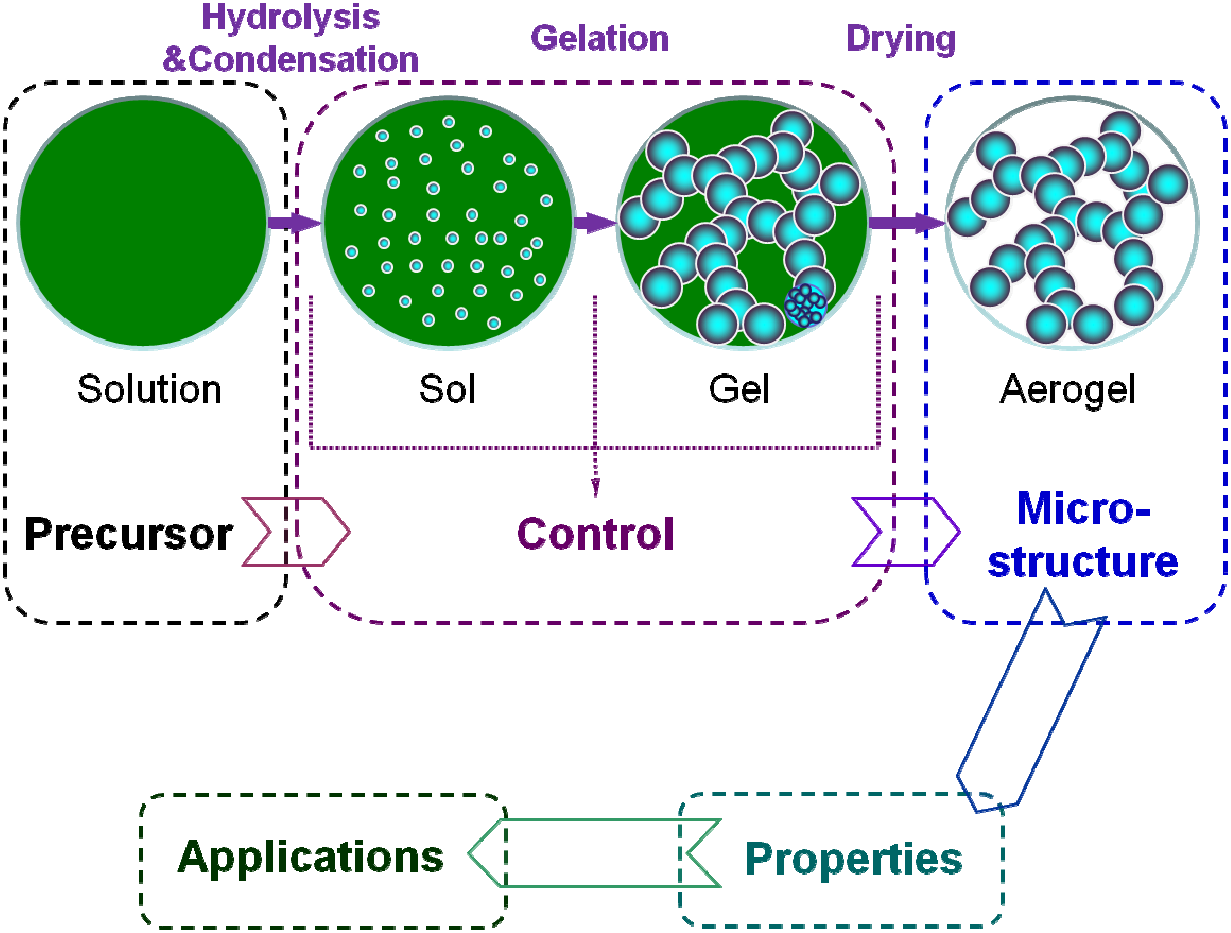
Basic research scheme for the aerogel.

The application design of the aerogel is based on its properties, which rely on the microstructure. Therefore, it is very important to realize the microstructure control during the preparation. Commonly, the preparation process of the aerogel includes following three key steps, as shown in the Figure 4 [71].

(i) Solution-sol transition: nanoscale sol particles are formed in the precursor solution spontaneously or catalyzed by the catalysts via hydrolysis and condensation reactions.
(ii) Sol-gel transition (gelation): the sol particles are crosslinked and hierarchically assembled into a wet gel with the coherent network.
(iii) Gel-aerogel transition (drying): the solvent inside the wet gel is replayed by the air without serious microstructure damage.

All three steps could determine the microstructure of the aerogel and affect its properties and applications.

The drying methods are mature and various, including high temperature supercritical fluid drying (SCFD), low temperature SCFD, natural drying, solvent-replaced ambient drying, surface-modified ambient drying, freezing drying and so on [57,72]. There would be, as it were, a suitable drying route to an aerogel, as long as a wet gel is formed (a key point in preparing the aerogel). Therefore, the solution-sol-gel transitions (sol-gel method) are focused instead on the drying method. The introduction of the drying processes is ignored on purpose, when referring to the preparation methods of different aerogels in this paper.

Although the basic idea is the same, the preparation of the aerogels with different composition or structure is much different. The details will be introduced in the following sections.

### 3.1. The Preparation of the Single-Component Aerogel

The preparation technique for the single-component aerogel is the base of the whole aerogel research. Theoretically, it is great possible to synthesize the composite aerogel if the preparation problem of corresponding single-component aerogels has been solved. It is scientifically significant but usually difficult to prepare a new kind of single-component aerogel because there is no relevant reference.

#### 3.1.1. Oxide-Based Aerogel

Oxide-based aerogel is the aerogel studied earliest and applied most widely. It is a most abundant class, which includes almost all species of aerogels mainly composed of metal-oxygen bonds. Given the importance, it will be introduced in more detail. Many concepts describing in this section may also facilitate the understanding of the following sections.

There are many methods for preparing the oxide-based aerogel, among which three methods are most versatile. The first method is the traditional sol-gel (TS) method. This method was first adopted to prepare silica aerogel by using organic precursor (Teichner’s group in 1968) [61]. After that, many including Al_2_O_3_, TiO_2_, ZrO_2_, Nb_2_O_5_, *etc.* were invented by using metal alkoxide as the precursor [16,58]. This method is still a most conventional and preferred route to the preparation of diverse aerogels now.

**Figure 5:**
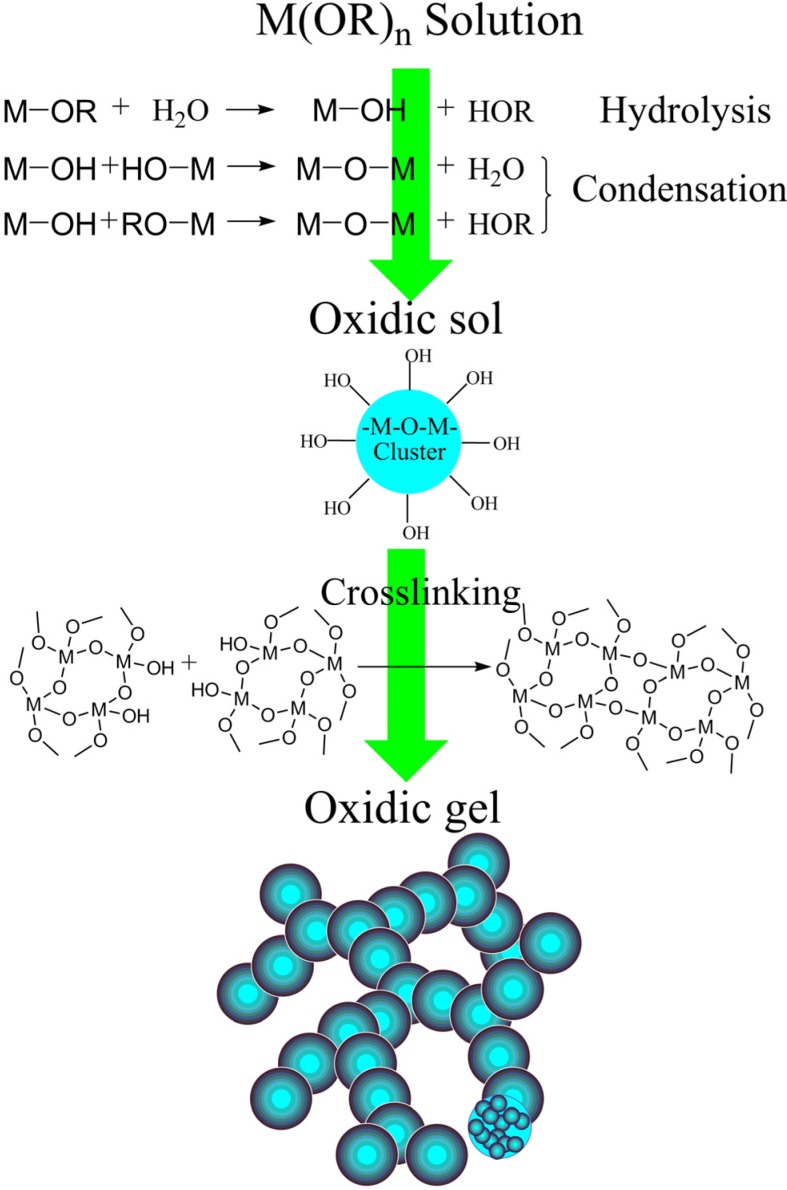
General scheme of traditional sol-gel method.

Basically, the transition from a solution to a gel relies on the hydrolysis and condensation reactions. As shown in Figure 5, the alkoxy group of metal alkoxide could react with the water to form a hydroxyl group, which is called “hydrolysis”. After the alkoxide is partly hydrolyzed, condensation occurs. Different metal atoms are bridged by an oxygen atom via a dehydration reaction between two separated hydroxyl groups or a dealcoholization reaction between a hydroxyl group and an alkoxy group.

The sol is only formed under suitable conditions determined by synthetic parameters. The fundamentals to form a sol are to adjust the reaction rates of cluster forming and cluster enlarging. Here we use the crystallography concepts of “nucleation” and “growth” to describe the forming and enlarging of the non-crystalline cluster, respectively, only in order to facilitate understanding. The audiences should notice that the concepts in this work and in the crystallography (to descript the forming and enlarging of crystalline nucleus) are obvious different.

Only if the nucleation rate is slower than or comparable to the growth rate, a sol consisting of separated and nanoscale particles could be formed; Otherwise precipitation tends to occur. This is easily understood. Relatively higher nucleation rate ensures the formation of more new-born clusters, and lower growth rate could keep the clusters small.

The nucleation rate is proportional to the ratio of supersaturation degree (concentration-saturation degree) to saturation, while the growth rate is related to the diffusion rate and supersaturation degree. For a homogeneous phase system, a high hydrolysis rate means a high hydrolyzing degree of the monomer (leads to high incompatibility and a low saturation degree) and a high supersaturation degree. All these result in a high nucleation rate. For the growth process, a high hydrolysis rate also leads to a high supersaturation, which accelerates the cluster growing. A low condensation rate will decrease the concentration difference near the surface of the newborn cluster, which leads to a low diffusion. Therefore, a high hydrolysis rate in the nucleation process and a low condensation rate could facilitate the formation of a colloid rather than a precipitate. In addition, to decrease the hydrolysis rate after nucleation is good for obtaining a stable colloid. According to this thought, an ultralight and structurally-fine aerogel was prepared via catalyzing the sol-gel reactions under acidic and alkaline conditions successively, instead of usual one-step acidic catalysis [73,74,75,76].

The sol derived from the alkoxide is a stable system in which nanoscale oxidic clusters (covered with active group like the hydroxyl) are dispersed by the solvent. Different clusters could be crosslinked via the condensations of their surface active groups. Finally, the gelation occurs when clusters are crosslinked throughout the whole system. The fractal 3d network is formed by the building block of the cluster via the hierarchical assembly, as shown in Figure 5.

A large number of studies have been done by adopting TS method especially for the preparation of silica, alumina or titania aerogels [58]. However, many other oxidic aerogels are not easy to prepare because of the extremely high condensation rate. The high hydrolysis rate, moreover, makes the sol-gel process uncontrollable. Thus, Gash developed a novel method (epoxide addition, EA method) by using inorganic salt as the precursor and epoxide as the mild catalyst [68]. The hydration and hydrolysis of the inorganic salt are relatively mild and easy to control. Its condensation rate strongly relies on the pH value. Different from the alkali, the epoxide reacts slowly with hydrogen ion under an acidic condition, which facilitates the condensation mildly. What’s more important, the reactivity variation of different inorganic salts is relatively slight compared with the alkoxide, which makes the EA method suitable for preparing many kinds of oxidic aerogels.

The epoxide addition method consists of three processes including hydration, hydrolysis and condensation. The metal salt in a water-contained solution mainly exists in the form of hydrated ion via a hydration reaction, as seen in the Formula (1).

(1) $$M^{n+}+xH_{2}O\to {\left[M{\left(H_{2}O\right)}_{x}\right]}^{n+}$$

The multistage hydrolysis reactions of hydrated ion takes place spontaneously, as shown in the Formulas (2) and (3). The reaction produces hydrogen ion and makes the solution partial acidic.

(2) $${\left[M{\left(H_{2}O\right)}_{x}\right]}^{n+}⇌{\left[M{\left(H_{2}O\right)}_{x-1}(\text{OH})\right]}^{(n-1)+}+H^{+}$$

(3) [M(H2O)x−y(OH)y](n−y)+[a⇌[M(H2O)x−y−1(OH)y+1](n−y−1)++H+

Epoxide could not increase the pH value rapidly like an alkali but slowly consumes hydrogen ion via a ring-opening nucleophilic addition reaction (Formula (4), taking propylene oxide and hydrochloric acid as an example). Thus, the hydrolysis balance turns right and produces the ion with the hydroxyl after adding the epoxide.



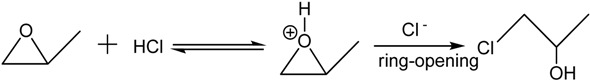
(4)


After that, dehydration condensation occurs by linking different hydroxylated ions with an oxygen bridge (forming an M–O–M bond, as shown in the Formula (5)). The condensation is rapid but limited by low rate of epoxide-catalyzed hydrolysis. Furthermore, the epoxide could maintain the initial condition acidic and facilitate the condensation mildly, compared with the alkaline condition. Both effects decrease the condensation rate and are good for forming a stable colloid.

(5) $$‑M‑\text{OH}+\text{HO}‑M‑\to ‑M‑O‑M‑+H_{2}O$$

Then, similar to the process introduced in TS method, the sol particles are formed, crosslinked and assembled into a gel skeleton.

The fundamental improvement is that the epoxide could lead to a relatively low rate of hydrolysis and condensation (especially for condensation rate), because the solution system could maintain in a low-pH environment for a long time. Iron oxide aerogel [23,68], nickel-based aerogel [21,24], alumina aerogel [18], stannic oxide aerogel [19], chromia aerogel [22,26], tantalum oxide *etc*. [25,77,78] have been prepared via EA method. Also, Gash *et al.* detailedly studied the influence of the parameters including salt type and epoxide type (1,2-epoxides including *cis*-2,3-epoxybutane, propylene oxide, 1,2-epoxybutane, glycidol, epichlorohydrin, epifluorohydrin and epibromohydrin, and 1,3-epoxides including trimethylene oxide and 3,3-dimethyloxetane) on the microstructure and properties of the iron oxide aerogels [20].

It is worth noting that the first divalent-element-based aerogel had been obtained by adding propylene oxide to a NiCl_2_·6H_2_O ethanolic solution. The gelation of the late 3d transition metal divalent ions is not easy to occur partially because “the aquocations of those ions were not sufficiently strong enough acids to induce” hydrolysis reaction [21]. What is more important, excluding the relatively weak interaction of bridge coordination (-oxo- or -hydroxyl-) or van der Waals force, the divalent ions only have two growth directions, which is difficult to build a stable 3D gel network.

In order to prepare copper-based aerogel, lots of attempts via EA method were carried out but all failed (only precipitates could be obtained) during 2005–2007. Thus, we started to try to add different kinds of soluble polymers, expecting to get a well-dispersed copper-based gel. Fortunately, we found that adding a small amount of polyacrylic acid (PAA) into an EA system was a good route. In 2007, we first reported the preparation and microstructure control of the Cu-based aerogels via an oral presentation and awarded an excellent prize in a national target meeting in China. Besides, we attended and posted a poster named as “preparation of monolithic copper oxide aerogels” in the 16th International Sol-Gel Conference. After that, we published the results and first named the method as the dispersed inorganic sol-gel (DIS) method in 2009 [27].

Interestingly, the method exhibits its versatility in aerogel preparation. Dong’s results showed that high-Z hydrated ions tend to form the microfissure derived from the M=O end, which makes the corresponding aerogels easy to crack [79,80,81]. The carboxyl in PAA could coordinate with the ions and restrain the M=O bond formation. Molybdena-based aerogel was prepared via both the coordination and electrostatic attraction effects of PAA [28].

In addition, this method was then used to prepare monolithic oxidic gels with diverse main elements including Li(I), Al(III), Ca(II), Ti(IV), Cr(III), Mn(II), Fe(III), Co(II), Ni(II), Cu(II), Zn(II), Zr(IV), Mo(IV), Cd(II), and Ta(IV). The DIS method exhibits incredibly wide applicability in preparation technology because almost all attempts use the same process and parameters, or even the same mixture ratio [26]. The polyacrylic acid could increase the nucleation rate because of the activation of the carboxyl site, and decrease the growth rate by decreasing the residual-ion concentration after rapid nucleation. Therefore, the reactivity differences among different elements are further decrease, which leads to a better versatility. Acting as both the dispersant and the template, PAA disperses the colloid system and guides the gel formation. Its carboxyl, moreover, provides extra crosslinking and restrains the formation of the terminal group, which could increase the formability of the gel.

The idea of the restricted-nucleation-growth mode in DIS method was then accepted by other researches. Detailed characterizations of copper-based aerogel and nickel-based aerogel via DIS method were reported by Bi *et al*. [24,82]. Kido *et al.* replaced the PAA with the poly(acrylamide) to prepare hierarchical iron-based xerogels via sol-gel process and phase separation [83]. In brief, the DIS method is now developing and needs further study.

#### 3.1.2. Organic Aerogel

Organic aerogel consists of resin-based aerogel and cellulous-based aerogel. The first resin-based aerogel was prepared by Pekala via Na_2_CO_3_-catalyzed polycondensation of resorcinol with formaldehyde (RF aerogel) in an aqueous solution [67]. In fact, the random network of RF gel is built by the homogeneous polymerization of resorcinol and formaldehyde in a large proportion of solvent (water).

The polymerization reaction consists of two steps: (1) addition reaction between resorcinol and formaldehyde to form hydroxymethyl resorcinol monomers (see Formula (6)); (2) –CH_2_– or –CH_2_OCH_2_– bridging polymerization between monomers, producing water/formaldehyde or water (Formulas (7) and (8)).



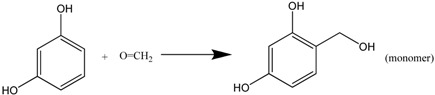
(6)

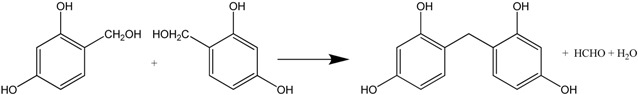
(7)

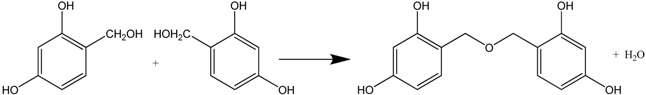
(8)


Continuous polymerization will produce RF clusters. The gelation occurs when the clusters crosslink throughout the whole colloid system.

This method could produce other kinds of resin-based aerogel [84,85,86,87]. Different catalysts could also be used to prepare RF aerogel [88,89,90,91]. However, the Na_2_CO_3_-catalyzed RF aerogel and corresponding sol-gel process are most widely adopted till now, because of its good formability, easy microstructure-controllability and adjustable properties.

However, the rate of these reactions in the room temperature is very slow. Normally, a multiple stage heating process is used to accelerate the gelation. In 2007, Mulik *et al.* developed a time-efficient acid-catalyzed method to prepare RF aerogels by using acetonitrile as the solvent [92]. The strong acid could accelerate the polycondensation obviously. The acetonitrile could disperse the colloid system to make it stable and prevent the colloid particles from agglomeration. This new system could produce very fine nanoscale RF skeleton under room condition. Low environment requirement and versatile solubility (of acetonitrile) make this method great potential to prepare functional composite aerogel.

The other kind of organic aerogel is cellulous-based aerogel. It was one of the “oldest” aerogels and the first organic aerogel, which was invented accompanying the birth of the silica aerogel [60]. Commonly, the cellulous gel is formed by dissolution and regeneration of cellulose in an aqueous or organic solvent [93]. Tan *et al.* found that the formability and mechanical properties of the cellulose aerogel (xerogel) obviously improved by crosslinking the gel skeletons with a crosslinker [94]. After that, the researches paid more attention to the applications and the preparation of the cellulose aerogel [95,96,97,98].

#### 3.1.3. Carbon Aerogel

**Figure 6:**
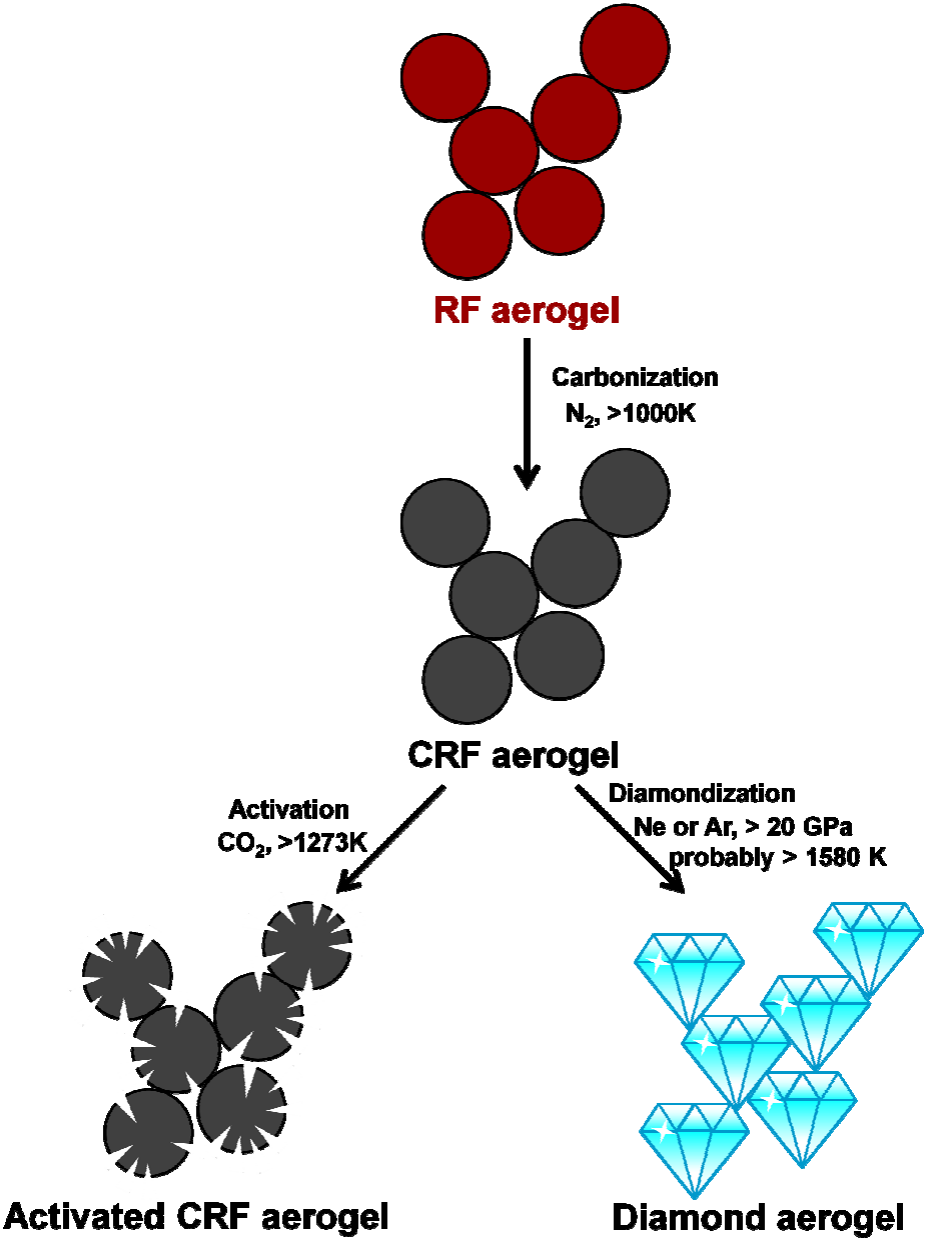
The scheme of the preparation of a carbonized RF (CRF) aerogel, activated CRF aerogel and diamond aerogel.

The first carbon aerogel was born in 1989 (Pekala) by carbonization of RF aerogel. It is usually considered as a kind of highly-porous amorphous-graphite-based foam. The basic idea of preparing carbonized RF (CRF) aerogel is to pyrolyze the high carbon-content template (RF aerogel) under high temperature (normally 800~1200 °C), ambient pressure and inert atmosphere. In 1996, Hanzawa *et al.* developed a route to a novel CRF aerogel with ultrahigh specific surface area by activating the carbon skeletons with carbon dioxide [99]. As shown in the Figure 6, carbon dioxide rather corrodes than activates the skeletons, creating more pores (mainly micropore). Much more active interfaces are created and useful for catalysis, adsorption, deionization and electrochemistry applications [100,101,102,103,104,105,106,107,108,109].

There is no fundamental improvement on the preparation of the CRF aerogel until 2011. Pauzauskie *et al.* crystallized the amorphous CRF aerogel template into the diamond aerogel under high pressure and high temperature by using a laser-heated diamond anvil cell (Figure 6) [110]. It is an amazing conversion that proves the aerogel could maintain its nanoscale skeleton via serious crystallization and phase-transition without distinctive densification. Diamond with the aerogel state was created and expected to sparkle soon.

Graphene-based aerogel is one of novel carbon aerogel, which was first prepared by Wang *et al.* in 2009 [50]. Graphene oxide solution was converted into the graphene aerogel by ultrasonic-induced gelation, drying and thermal reduction. In 2010, Worsley *et al.* reported the route to graphene-based aerogel by carbonizing the RF-crosslinked graphene-oxide aerogel [49]. In 2011, Zhang *et al.* reported a much simple method to prepare “pure” graphene aerogel via reduction/self-crosslinking of graphene oxide dispersion induced by L-ascrobic acid and drying of the wet graphene gel [47]. This method is significant because no additional pyrolysis treatment is needed. In addition, in 2011, Worsley *et al.* made comparisons between pure graphene aerogel and graphene-CRF aerogel [111]. This subset remains hot and focused now.

Another kind of interesting carbon aerogel is carbon nanotube (CNT) aerogel. It was first created in 2007 by dispersing CNT into a surfactant-contained solution under sonication, then gelling and drying [112]. The aerogel could be further enhanced by polyvinyl alcohol. In 2009, Aliev *et al.* reported the synthesis of CNT aerogel muscles by drawing from straight sidewalls of multiwall nanotube forests [43]. Different from almost all the other aerogels, its raw material (CNT forests) was prepared via catalytic chemical vapor deposition but not sol-gel process. Another “dry synthetic method” to prepare carbon-based aerogel (aerographite) is to deposit nano-structured graphite on ZnO network templates (chemical vapor deposition), reduce ZnO to metallic Zn in hydrogen atmosphere and subsequently sublimate Zn under high temperature [113]. The resultant possesses ultralow density (<0.2 mg/cc) and outstanding mechanical properties, which may get wide applications.

#### 3.1.4. Chalcogenide Aerogel

In 1997, Gacoin *et al.* reported the preparation of the wet gel and xerogel of CdS via the reverse micelle growth, capping, segregation, redispersion and crosslinking processes [114]. In 2004, Brock’s group synthesized the first chalcogenide aerogel (CdS) by using a similar method and a low-temperature SCFD technique [35]. In 2005, Brock’s group published their work on the preparation of diverse chalcogenide aerogels in Science and aroused wide interests [34]. In 2007, Bag *et al.* and Brock’s group reported (also in Science) the preparations of Pt^2+^/chalcogenide-cluster aerogels and Hg^2+^-modified Pt^2+^/chalcogenide-cluster aerogels, respectively, in order to adjust the band gap of the aerogels [115]. In addition, in 2007, Brock’s group reported the reaction mechanism in detail [116]. After that, various chalcogenide aerogels were reported including direct-synthesized aerogels and ion-exchanged aerogels [29,30,31,32,117,118,119].

The basic method to prepare chalcogenide aerogel is ingenious. In a homogeneous system, it is difficult for the chalcogenide to form a stable gel skeleton because of its weak crosslinking ability and rapid growth rate. Instead, precipitates are commonly formed. The growth of the primary chalcogenide nanoparticles (NPs) is restricted inside the small water-phase region of the reverse micelle system. To cap the NPs could prevent them from agglomeration. After redispersion, the capped groups are oxidized gradually by an oxidant (normally H_2_O_2_). The separated NPs crosslink and are finally conversed into a stable gel. This idea has broad significance in the preparation of the other aerogels, which are difficult to form in a homogeneous system.

#### 3.1.5. Other Single-Component Aerogel

The other single-component aerogels certainly include some aerogels derived from natural materials, like gelatin, agar, egg albumin and rubber, and first created by Kistler [60]. However, other artificially-synthesized aerogels with pure composition and controllable microstructure will be focused in this section.

For example, a silicon imidonitride aerogel with the Si-N-Si based skeleton is interesting and derived from Si(NHMe)_4_ [120].

The carbide-base aerogel and metal-based aerogel could be prepared via a carbothermal conversion. In 2010, Leventis *et al.* first prepared the SiC aerogel via a click reaction of the silica aerogel template with coated polyacrylonitrile [53]. Worsley *et al.* conversed the silica-coated carbon aerogel into SiC/C composite aerogel via a high-temperature carbothermal process [121]. Leventis *et al.* further demonstrated a versatile route to diverse porous metals and carbides by carbothermal treating of metal oxide/RF aerogel [122]. Furthermore, many papers concerning SiC materials were published after that [51,123,124,125,126].

It is worth noting that Jung *et al.* developed a facile route for 3D aerogels from diverse 1D or 2D nanoscale building blocks including Ag nanowires, MnO_2_ nanowires, single-walled carbon nanotubes, MoS_2_ nanosheets, graphene and h-BN nanosheets [127]. The preparation method consists of three processes, including (i) dispersing the building blocks into a large amount of solvent with or without adding surfactants, (ii) slowly evaporating the suspension to promote gelation and (iii) supercritical fluid drying. Much more kinds of novel aerogels are expected to be prepared via this method because the build blocks are much easier to be synthesized than common aerogels.

Our group developed a simple way to prepare the SiC aerogel via the Mg-catalyzed low-temperature treatment of RF/silica aerogel template [51]. The template is prepared by co-gelation of successively addition of RF sol and silica precursor. Magnesium vapor possesses stronger reducibility than the carbon, which may synthesize more kinds of carbide or even metal aerogels. According to this idea, pure Si (silicon) aerogel was prepared by direct magnesiothermic reducing the SiO_2_ aerogel [52].

### 3.2. The Preparation of the Composite Aerogel

The preparation of a single-component aerogel is the fundamental aim of aerogel research. Theoretically, any of composite aerogels could be prepared if only the corresponding single-component aerogels could be prepared. It seems that to prepare a composite aerogel is a technical problem rather than a scientific problem. However, this fact is not so encouraging. Details will be discussed in the following sections.

#### 3.2.1. Multi-Composition Aerogel

Here the multi-composition aerogel refers to the aerogel with interpenetrating network of different chemical compositions. In other words, the aerogel with the dopant, which is not constructed inside the gel skeleton is not introduced in this section.

The crux of the preparation of a rigid multi-composition aerogel is to match any of synthetic parameters of the corresponding single-component aerogels, including solvents, pH values, catalysts, nucleation/growth rates, temperatures, pressures and so on. For example, metal oxide/silica aerogels combine the function of metal oxide and the rigid microstructure of the silica aerogel, however, the composite aerogels with high metal-oxide content are not easy to prepare via TS method, because of the mismatch of hydrolysis/condensation rates between metal alkoxide and silicon alkoxide. In 2003~2004, Gash’s group solved this problem and developed a versatile route to metal-silicon mixed oxide aerogels by co-gelling the precursors of inorganic metal salts and TMOS via EA method [128,129]. The metal oxide in the composite aerogels may even be a major phase. Instead of successively adding epoxide and fluohydric acid (HF, efficient catalyst for TMOS gelation), only epoxide was added to accelerate the slow co-gelation because of the mismatch (reaction) between epoxide and HF. Our group further developed a method to form a composite gel rapidly via pre-reaction of TMOS with epoxide and co-gelation of silica/metal oxide in the co-solvent of acetonitrile [130].

The epoxide addition method is also useful for preparing multi-metal-oxide aerogels. According to this idea, CuO-NiO aerogel and indium-tin oxide (ITO) aerogel were synthesized to be designed as the catalyst and conductive oxide, respectively [131,132].

Metal oxide-containing RF aerogel is a robust template for preparing metal oxide/carbon aerogel, metal/carbon aerogel and carbide aerogel. The method of the impregnation of functionalized RF network with metal salt solution leads to a relatively low metal content. In 2009, Leventis *et al.* found a novel system to prepared CuO/RF aerogels by using N,N-Dimethylformamide (DMF) as co-solvent and acid catalysis RF sol as RF source (from the method of reference [92]) [133]. The interpenetrating structure could ensure high content of metal oxide and wide potential to prepare the other metal oxide/RF composite aerogel.

#### 3.2.2. Gradient Aerogel

In 1992, Fricke’s group prepared the first density-gradient aerogel by making a homogeneous aerogel shrink different in a temperature-gradient environment, but the gradient was not very high [134]. The structure-distributed aerogels prepared by layer-by-layer sheet-pasting method, layer-by-layer gelation method and layered sol co-gelation method should be assigned to graded rather than gradient aerogels in a narrow sense [135,136]. The high gradient aerogel was successfully prepared by Jet Propulsion Laboratory by co-gelation of gradient-mixed silica sol [36,37,39]. In addition, we prepared the first Chinese gradient aerogel and successfully collected the high-velocity (>3 km/s) particles with the capture depth of only several millimeters [137].

Our other attempt to prepare ultrathin graded RF-based aerogel was successful, by combining the methods of micro molding and layer-by-layer gelation. This aerogel has been used as a reservoir to study the equation of state of the aluminum under low temperature and high pressure, and will be reported soon.

#### 3.2.3. Micro-/Nano-Composite Aerogel

The aerogel is a state of matter with many unique properties. The probably only disadvantage of the aerogel is its weak skeleton whose Young’s modulus is as low as ~10^4^ Pa and yield strength is just several kPa. What’s more important, the aerogel is extremely fragile, which limits its development in practical applications. The friable nature could not be improved fundamentally via the strengthening of its skeleton microstructure by only adjusting the synthetic parameters, because the aerogel possesses ultralow solid content and numerous defects. In 1999, Morris *et al.* reported a flexible rout to composite aerogels by using about-to-gel silica sol as a nanoglue [138]. Many researches adopted the idea to reinforce the nanoporous aerogel with the micron-scale fiber or fiber felt, so did Aspen Inc., Cabot Corporation and Nano High-tech Co. Ltd. in China [57,139,140]. Micron-scale powder is another type of additive, which could decrease the infrared radiation heat transfer, improve thermal stability or be used as the reductant [141,142,143,144].

To cap a silica aerogel skeleton with resin or interpenetrate the networks of silica aerogel and resin is also an efficient way to strengthen the aerogel [145,146,147,148,149,150,151,152,153]. The surface modification of the aerogel could facilitate the combination and make the composite aerogel homogeneous. By the way, the “inverse” idea to fill the aerogel powder into resins also improves the mechanical and thermal properties of the resins noticeably [154].

## 4. Research Tendency

As shown in Figure 2, research contents of the aerogel consists of single-component aerogel research and composite aerogel research, similar to the Chinese Tai Chi composed of Yang and Yin, respectively. The studies on the single-component aerogel are fundamental, scientific and potentially applicable, while the studies on the composite aerogel are practical, technical and direct applicable. It is equally important to prepare novel single-component aerogels, design composite aerogels or apply the aerogels in industry.

### 4.1. The Preparation of Novel Single-Component Aerogels

To create a single-component aerogel with the novel composition is relatively difficult but fundamentally valuable. A series of new aerogels including chalcogenide aerogel, CNT aerogel, graphene aerogel, diamond aerogel, silicon imidonitride aerogel, *etc.* were successively invented in the 21st century. The next booming class may be carbide aerogel or single-element (mainly metal) aerogel. Leventis *et al.* has laid a good foundation for preparing them via the carbothermal reduction [122]. The metal oxide/RF aerogel templates for preparing carbide aerogel or single-element aerogel could be various and controllable. On one hand, almost all kinds of stable metal-oxide aerogels could be facilely prepared via DIS or EA method. On the other hand, acid-catalyzed RF sol (also developed by Leventis *et al.*) with a suitable solvent, is easy to interpenetrate with metal oxide networks. Thus, potentially more kinds of carbide aerogels or single-element aerogels could be prepared via the magnesiothermic reduction. The hope is that increasingly, new matters could fill the aerogel state to make this state more acceptable.

### 4.2. Material Design of Composite Aerogels

As mention in previous sections, a great variety of the aerogels have been prepared before, which provides plenty of techniques to design and prepare composite aerogels with practical and smart functions. For example, ITO aerogel, YSZ (yttria-stabilized zirconia) aerogel and binary oxide aerogel have been designed as oxide conductor and catalyst [131,132,155,156]. Carbonized-RF aerogel is, all the while, regarded as one of highly-efficient electrode materials for supercapacitor or capacitive deionization. Recently, Chien *et al.* reported an excellent supercapacitor with ultrahigh specific capacitance (~1700 F/g, much high than the value of single carbon aerogel of ~200 F/g), high rate capability and outstanding cycling stability, by using nickel cobaltite/carbon aerogel as the electrode [157]. Thus, to use metal oxide/CRF composite aerogel is presumably a good way to improve the capacitive properties considerably [158,159].

In addition, the aerogel is versatile for the high-energy physics experiments. For example, ultralight metal oxide/silica aerogels could greatly increase the efficiency for laser–X-ray conversion [160,161,162,163,164,165,166,167]. The efficiency may be further improved via a concentration gradient design. Ultrathin graded aerogel could be used to study the equation of state of the other matters under low temperature and high pressure. Density-adjustable bilayer perturbation aerogel could be used as potential target in hydrodynamic instability experiment [168]. Optimized gradient curve may further improve the efficiency for high-velocity particle collection. Many other applications could be designed according to the requirements and preparation techniques.

### 4.3. Industrial Application of the Aerogels

Compared with the other applications, industrial applications benefit human beings directly. However, because of the bad formability or mechanical properties, most of the aerogels are difficult to use in industry at the present stage. Moreover, low yield and high cost limit the commercialization of the aerogels. Thus, the aerogels used in high-energy physics and space exploration often cannot be direct applied in industry. The silica aerogel, which is the oldest and most mature aerogel, exhibits tremendous commercial and civil value. The Chinese government has made a national guideline in the 12th Five-Year Plan to use the aerogel as the thermal insulation for buildings. Fiber-reinforced silica aerogel complying with Chinese national standards of non-ignitable, waterproofness, strength and thermal insulation property is required for external thermal insulation of outer wall. Two alternative drying methods including surface-modified ambient drying and quasicontinuous-producing CO_2_ SCF drying are available. Ambient drying needs an expensive and recyclable silane-based modifier, while SCFD holds potential safety hazard derived from high pressure. The final choice will be made mainly according to the evaluations of environmental effect, productive efficiency, cost, safety and properties. We hope to see plenty of green buildings wearing efficiently-insulated and “fashionable” aerogel clothes in the near future.

## 5. Conclusions

The categories, applications and preparation methods of the aerogel are various after developed for ~80 years. The unique characteristics and diverse chemical compositions make the aerogel recognized as a state of matter. In this review, the symbolic preparation methods of single-component aerogels and composite aerogels including oxidic aerogel, organic aerogel, carbon aerogel, chalcogenide aerogel, multi-composition aerogel, gradient aerogel, micro-/nano- composite aerogel and many other kinds of aerogels were introduced in detail and expected to inspire the audiences to create new kind of aerogel or make novel designs for the applications. There are three upsurges of the aerogel research, among which the first two upsurges were driven by a few marked technologies but the third one was motivated by plenty of new technologies after entering the 21st century. The aerogel research is still developing and booming now. Hopes more and more researchers focus on the preparation of novel single-component aerogels, material design of composite aerogels and industrial application, to give this state of matter a bright future.
